# Emphysematous Pyelonephritis and Emphysematous Cholecystitis: A Result of Uncontrolled Type 2 Diabetes

**DOI:** 10.31486/toj.20.0126

**Published:** 2021

**Authors:** Fred Alain Montelongo-Rodríguez, José Iván Robles-Torres, Jesús García-Saucedo, Efraín Ruíz-Galindo, Carlos Pacheco-Molina, Lauro Salvador Gómez-Guerra

**Affiliations:** ^1^ Department of Urology, Hospital Universitario Dr. José Eleuterio González, Universidad Autónoma de Nuevo León, Monterrey, Nuevo León, México; ^2^ Department of Surgery, Hospital Universitario Dr. José Eleuterio González, Universidad Autónoma de Nuevo León, Monterrey, Nuevo León, México

**Keywords:** *Diabetes mellitus*, *emphysematous cholecystitis*, *emphysematous pyelonephritis*

## Abstract

**Background:** Emphysematous pyelonephritis (EPN) is a life-threatening necrotizing infection that results in the presence of gas in the renal parenchyma, collecting system, and surrounding tissues. Up to 95% of patients with EPN have underlying uncontrolled diabetes mellitus. Emphysematous cholecystitis (EC) is a necrotizing infection defined by the presence of gas in the gallbladder. Concurrent presentation of EPN and EC is limited to anecdotal cases in the literature.

**Case Report:** We present the case of a 44-year-old female who arrived at the emergency department with signs of septic shock and diffuse abdominal pain. Diagnosis of EPN and EC was confirmed. Because the patient did not improve after aggressive medical therapy and percutaneous drainage and cholecystostomy, she was taken to surgery for emergency nephrectomy and cholecystectomy.

**Conclusion:** In unusually extensive and severe cases of EPN, medical and minimally invasive procedures are not enough to control the infection. More aggressive management, including emergency surgery, should be implemented in selected patients who present with refractory septic shock associated with extensive disease.

## INTRODUCTION

Emphysematous pyelonephritis (EPN) is a life-threatening infection characterized by the presence of gas in the renal parenchyma, collecting system, and surrounding tissues attributed to an acute necrotizing process.^1^ Up to 95% of patients with EPN have uncontrolled diabetes mellitus.^1,2^ Emphysematous cholecystitis (EC) is a necrotizing infection defined by the presence of gas in the lumen or wall of the gallbladder. Concurrent presentation of EPN and EC is limited to anecdotal cases in the literature.^3-6^ We present the case of a 44-year-old female who presented with signs of septic shock and diffuse abdominal pain. The diagnosis of EPN and EC was confirmed.

## CASE REPORT

A 44-year-old female with no relevant medical history presented to the emergency department with a 1-week history of abdominal pain, nausea, shortness of breath, and fever. She had a history of recurrent urinary tract infections and denied history of biliary colic. On examination, she was febrile, agitated, and confused. Vital signs upon arrival were heart rate of 110/min, blood pressure of 80/60 mmHg, respiratory rate of 26/min, oxygen saturation of 93%, and temperature of 101.3 °F. She reported significant bloating and diffuse abdominal pain. Laboratory tests showed hemoglobin of 11.3 g/dL (reference range, 12.2-18.1 g/dL), leukocyte count of 5.3 K/uL (reference range, 4.0-11.0 K/uL), glucose of 400 mg/dL (reference range, 60-100 mg/dL), and serum creatinine of 3.7 mg/dL (reference range, 0.6-1.4 mg/dL). Arterial blood gas analysis was consistent with metabolic acidosis (pH 7.15, HCO_3_ 18 mEq/L, PaO_2_ 89 mmHg). Urinalysis showed positive leukocyte esterase and nitrites. Urine microscopy revealed leukocyturia, hematuria, and moderate bacteriuria. The patient denied a history of diabetes mellitus.

**Figure 1.**
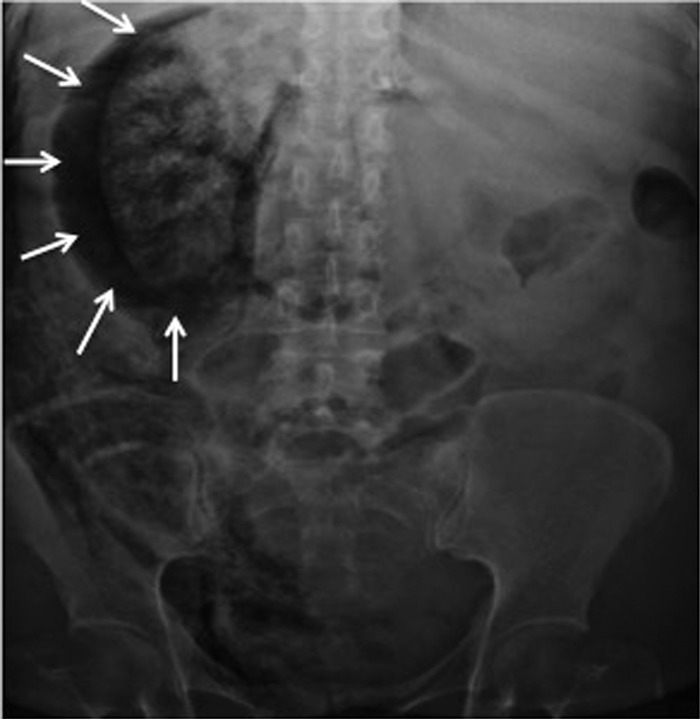
Abdominal x-ray shows gas distributed in the topography of the right kidney (arrows).

**Figure 2.**
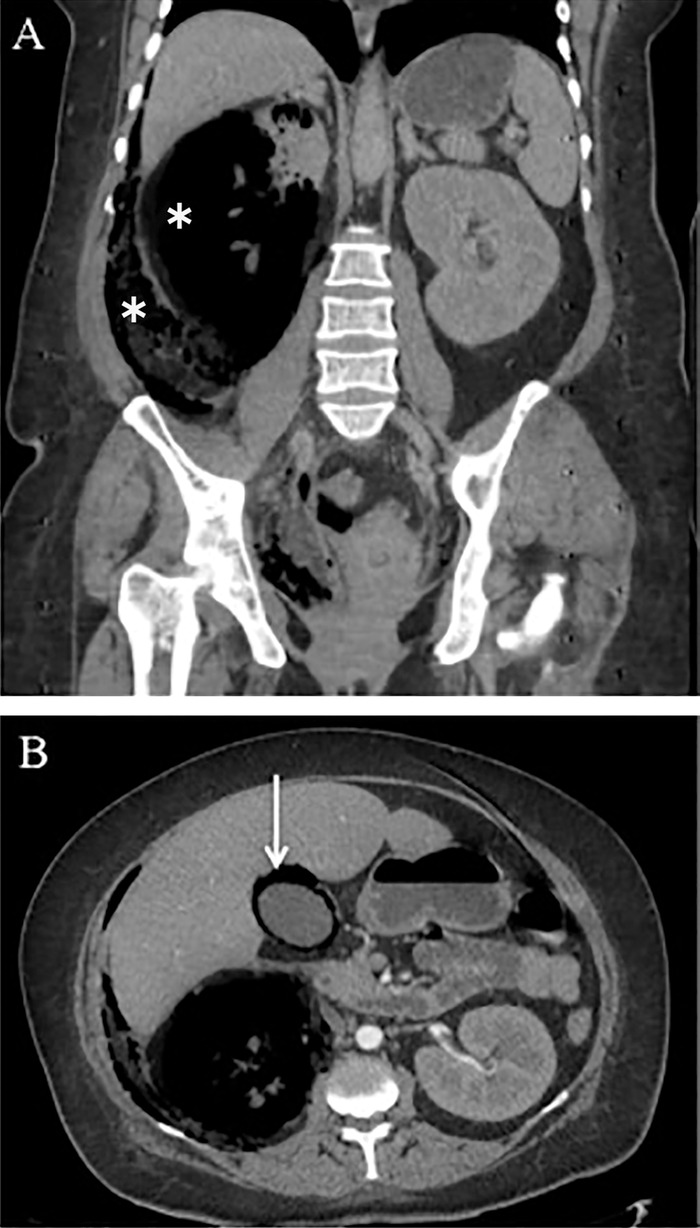
(A) Coronal view of contrast-enhanced computed tomography (CT) scan shows extensive parenchymal destruction of the right kidney (top asterisk) and gas extending through the retroperitoneum (bottom asterisk). (B) Axial view of contrast-enhanced CT scan shows intramural gas in the gallbladder (arrow).

Abdominal x-ray revealed gas over the right renal topography (Figure 1). Contrast-enhanced computed tomography scan of the abdomen revealed destruction of the right renal parenchyma, gas extending from the retroperitoneum to the retropubic space, and gas within the gallbladder wall (Figure 2). Gas was also seen in the mediastinum. The diagnosis of EPN, EC, and pneumomediastinum was confirmed.

Empirical intravenous (IV) broad-spectrum antibiotics were administered. Imipenem and cilastatin with a renal dosage adjustment (250 mg IV every 12 hours) were initiated, along with fluid resuscitation with a crystalloid solution of sodium chloride at 0.9% every 8 hours. Percutaneous drainage of the kidney and gallbladder was performed 24 hours after arrival. A total of 140 mL of purulent material from the perinephric puncture was drained, as well as cloudy bile fluid from the percutaneous gallbladder puncture. Forty-eight hours later, the patient showed no clinical improvement, with persistent altered state of consciousness, high fever despite the medical management, and an increase in leukocyte count to 18.2 K/uL.

The patient was taken to the operating room for cholecystectomy and nephrectomy. A midline incision was made. The patient's gallbladder was enlarged, had a necrotic appearance, was extremely friable, and was attached to the liver. The kidney was completely destroyed and had a dark brown and necrotic appearance. At the time of renal dissection, friable perinephric tissue was found, as well as a fibrotic renal hilum, which made removal and hemostasis technically difficult. Total blood loss was 3.5 L, and the patient required 3 units of blood and 2 platelet units. A closed drain was placed in the perinephric space. Microscopy reported extensive proliferation of gram-negative bacilli in the renal parenchyma with extensive perinephric involvement and acute and chronic inflammatory changes. The gallbladder had embolic microabscesses and chronic inflammatory changes. Microbiologic analysis reported the same strain of *Escherichia coli* from urine, right kidney, perinephric exudate, blood, and gallbladder, suggesting hematogenous spread of the infection.

The patient required a 15-day hospital stay, with 10 days in the intensive care unit. She was discharged when her leukocyte count normalized. The drain was removed before discharge. Acute kidney injury was managed with a proper fluid replacement, and glycemic levels were within normal ranges achieved by IV insulin infusion administration. The patient currently has adequate glycemic control managed with intermediate and rapid insulin, as well as a diet managed by the nutrition team of our hospital.

## DISCUSSION

EPN is a urologic emergency that usually presents with signs of sepsis and often requires intensive care unit management.^7^ Factors involved in the pathogenesis of EPN are gas-forming bacterial infection, high tissue glucose level, impaired tissue perfusion, and immunosuppression.^8^ Gram-negative bacteria such as *E coli* cause tissue necrosis and gas formation by fermentation of glucose and lactate, given the facultative anaerobic characteristic of these bacteria.^1^

EC is an uncommon life-threatening condition with an approximate 5-times greater risk of perforation of the gallbladder compared to acute non-EC. Compared to EPN, a diagnosis of EC is related to diabetes mellitus in only 20% to 30% of patients. Some authors have suggested hematogenous spread of the gas-producing organisms.^6^

EPN can progress rapidly if therapeutic measures are not applied early. Gas and necrosis can spread to surrounding organs and the entire circulatory system by hematogenous dissemination.^9^ The same microorganism was found in our patient's blood, urine, and gallbladder cultures, suggesting that the etiology of EC was septic embolism related to a hematogenous spread of gas-forming microorganisms from the kidney. This route was the most likely cause of the spread of infection to the gallbladder.^4^

With their clinical-radiologic classification of EPN, Huang and Tseng demonstrated that mortality increases as EPN severity increases.^9^ Huang and Tseng identify 4 EPN classifications: (1) class 1, gas in the collecting system only; (2) class 2, gas in the renal parenchyma without extension to the extrarenal space; (3) class 3A, extension of gas or abscess to the perirenal or pararenal space; and (4) class 3B, bilateral or solitary kidney with EPN. Our patient had class 3B EPN with extensive dissemination. Furthermore, Huang and Tseng demonstrated that thrombocytopenia, acute kidney injury, altered state of consciousness, and septic shock were factors of poor prognosis and high mortality.^9^ Our patient presented with an unusual extension of the disease, with multiple risk factors for poor prognosis such as hypotension, altered state of consciousness, and acute renal injury.

To the best of our knowledge, this case is the fifth report of concurrent EPN and EC. The patients reported by Zaragoza et al and Lee and Jeffrey underwent emergency nephrectomy.^5,6^ Bhansali et al and Holbrook and Kalaidina chose medical management and delayed cholecystectomy, but these patients presented with minimal renal extension.^3,4^ Our patient had similarities to the case reported by Zaragoza et al; both patients presented with extensive emphysematous disease. The patient reported by Zaragoza et al died from multiorgan failure. The difference between the 2 cases is that the patient reported by Zaragoza et al was surgically managed without previous intervention (eg, percutaneous drainage) compared to our patient who was previously drained. Aboumarzouk et al found that percutaneous drainage and medical management alone were associated with a lower mortality rate compared to emergency nephrectomy.^10^ In contrast, the patient reported by Lee and Jeffrey had a good outcome, despite having emergency nephrectomy without previous drainage, but emphysematous disease was less extensive.^6^

## CONCLUSION

In unusually extensive and severe cases of EPN, medical and minimally invasive procedures are not enough to control the infection. More aggressive management, including emergency surgery, should be implemented in selected patients who present with refractory septic shock associated with extensive disease.

## Notes

*This article meets the Accreditation Council for Graduate Medical Education and the American Board of Medical Specialties Maintenance of Certification competencies for Patient Care and Medical Knowledge.*
